# The Association of Serum hsCRP and Urinary Alpha1-Microglobulin in Patients with Type 2 Diabetes Mellitus

**DOI:** 10.1155/2019/6364390

**Published:** 2019-06-09

**Authors:** Xiaohua Wan, Lin Zhang, Haitong Gu, Shenglai Wang, Xiangyi Liu

**Affiliations:** ^1^Department of Clinical Laboratory, Beijing Tongren Hospital, Capital Medical University, Beijing, 100730, China; ^2^Department of Endocrinology, Beijing Tongren Hospital, Capital Medical University, Beijing, 100730, China; ^3^Beijing Key Laboratory of Diabetes Research and Care, Beijing, 100730, China; ^4^Beijing Diabetes Institute, Beijing, 100730, China

## Abstract

This study aimed to investigate the association of serum hsCRP and urinary A1MG in patients with T2DM. Numerous investigations have proven that serum hypersensitive C-reactive protein (hsCRP) concentration in patients with type 2 diabetes mellitus (T2DM) is increased. Also, increased urinary alpha-1 microglobulin (A1MG) can be an early sign of renal damage, primarily on the proximal tubules in T2DM. Little information is available with respect to the associations of serum hsCRP levels and urinary A1MG in T2DM. A total of 520 patients with T2DM were recruited to participate in this study. Serum hsCRP and UA1MG (urinary alpha1-microglobulin to creatinine ratio), UACR (urinary microalbumin to creatinine ratio), UIGG (urinary immunoglobulin G to creatinine ratio), and UTRF (urinary transferrin to creatinine ratio) were obtained. The association of serum hsCRP level and each urinary protein parameter was analyzed by using the regression analysis, respectively. LnhsCRP was positively associated with the lnUA1MG in all three linear regression models (adjusted *β* in model 3=0.122, SE=0.027, P<0.001). Furthermore, the high hsCRP group (hsCRP > 3mg/L) was associated with increasing risk of high UA1MG (adjusted OR in model 3=1.610, 95% CI 1.037–2.499, P=0.034) by logistic regression. This study suggests that serum hsCRP levels independently associate with UA1MG in patients with T2DM. Further research is warranted to elucidate these interactions.

## 1. Introduction

Type 2 diabetes mellitus (T2DM) is one of the most common chronic diseases, and its incidence continues to increase. T2DM causes a series of physiological and pathological changes in the body and chronic lesions in lung, heart, brain, kidney, nerve, and other organs and even leads to functional defects and failure [1–3]. Novel findings are suggesting that activation of innate immune system as well as low-grade inflammation may have an important role in the pathogenesis of T2DM [4]. It has been demonstrated that systemic low-grade inflammation is associated with an increased risk for the development of T2DM [5].

Hypersensitive C-reactive protein (hsCRP) is an acute phase protein and it represents extremely sensitive systemic marker of inflammation and tissue damage. Recent studies indicate that hsCRP might be activator of nonspecific immunity and modulator of specific immunity [5]. Numerous investigations have proven that serum hsCRP concentration in patients with T2DM is increased. It has been demonstrated that determination of serum hsCRP levels may predict possibility for development of T2DM [5]. Most of the authors consider that increased serum hsCRP concentration might be the reflection of present low-grade inflammation which precedes the development of T2DM [5, 6]. Thorand et al. [7] have shown that hsCRP is a significant predictor for T2DM development in middle aged men independent of classical risk factors such as triglycerides level, body mass index, fasting glucose, or smoking.

Diabetic nephropathy (DN) has been widely recognized as a common complication of T2DM, which may further progress into end-stage renal disease and premature mortality[8, 9]. Growing evidence indicates that immunologic and inflammatory mechanisms play a significant role in disease development and progression in DN. Studies suggest that individuals who develop diabetic kidney disease have low-grade inflammation years before the onset of the disease [1]. Several human studies support these findings, and several cross-sectional studies have reported that high levels of inflammatory markers, such as IL-6, fibrinogen, or hsCRP, are associated with diabetic nephropathy in patients with diabetes [10]. HsCRP itself was induced by high level of glucose, which then promoted renal inflammation, so hsCRP may serve as an inflammatory mediator of high glucose levels to promote the diabetic renal inflammation [11, 12].

A number of key biomarkers present in the urine have been identified that reflect kidney injury at specific sites along the nephron, including glomerular/podocyte damage and tubular damage, oxidative stress, inflammation, and activation of the intrarenal renin-angiotensin system [1]. Alpha-1 microglobulin (A1MG) is a small molecular weight protein (27 kDa) present in various body fluids. In the healthy kidney, it passes freely through the glomerular membranes, and about 99% is reabsorbed and catabolized by the proximal tubular cells [9]. When the reabsorption function of renal tubules is failed, the output volume of A1MG will increase. Therefore, increased urinary A1MG can be an early sign of renal damage, primarily on the proximal tubules [13–16]. Also, urinary microalbuminuria (mAlb), urinary immunoglobulin G (IGG), and urinary transferrin (TRF) were considered markers of glomerular dysfunction [17–20]. Clinically, UA1MG (urinary alpha1-microglobulin to creatinine ratio), UACR (urinary microalbumin to creatinine ratio), UIGG (urinary immunoglobulin G to creatinine ratio), and UTRF (urinary transferrin to creatinine ratio) were often used as the key biomarkers to evaluate the severity of diabetic nephropathy.

However, because of the nature of a cross-sectional study, the direct causality between high levels of inflammatory markers and the panel of urinary protein parameters is unknown. To the best of our knowledge, little information is available with respect to the association of serum hsCRP level and UA1MG in T2DM.

Therefore, we studied a cohort of Chinese patients with type 2 diabetes from a single-center registry to determine the prospective association between baseline serum hsCRP concentration and the panel of urinary protein parameters (UA1MG, UACR, UIGG, and UTRF).

## 2. Materials and Methods

### 2.1. Patients

We recruited 520 inpatients with T2DM at the Department of Endocrinology of Beijing Tongren Hospital, Capital Medical University, in Beijing from August 2017 to September 2018. Inclusion criteria were clinically diagnosed T2DM longer than 5 years. T2DM was diagnosed on the basis of the World Health Organization criteria [21]. Exclusion criteria were presence of those with type 1 diabetes, those with nephrotic syndrome, acute kidney injury, acute infection, and malignancy including gastric cancer, active gastrointestinal diseases including gastroenteritis and peptic ulcers, or liver cirrhosis, and history of rapidly progressive renal failure, glomerulonephritis, and polycystic kidney disease.

We reviewed detailed demographic data, biochemical data, and clinical and treatment histories from patient medical records. All patients were informed of the purpose of the study and written consent was obtained. The study was approved by the Institutional Review Board of Beijing Tongren Hospital, Capital Medical University.

### 2.2. Anthropometric Measurements

Basic anthropometric measurements, body height (cm) and body weight (kg), were obtained. Body mass index (BMI) was calculated by body weight (in kilograms) divided by height (in meters) squared [22]. Each patient's arterial blood pressure was measured by a physician after a 10-minute resting period to obtain the systolic and diastolic blood pressures (SBP and DBP, respectively).

The Chronic Kidney Disease Epidemiology Collaboration (CKD-Epi) equation was used to estimate glomerular filtration rate (eGFR CKD-Epi) [23, 24]. Insulin resistance status was evaluated by the homeostasis model assessment-insulin resistance (HOMA-IR) index. The HOMA-IR was calculated using the formula [fasting insulin (uIU/mL) × fasting blood glucose (mmol/L)]/22.5. The HOMA-IR score was available in only 427 patients [25].

### 2.3. Sample Collection and Laboratory Methods

After at least 8 h of an overnight fasting, a venous blood sample was obtained from the forearm of each participant. Participants were requested to provide two blood samples, one for whole blood in K_2_ EDTA for HbA1c (glycosylated hemoglobin) determination and the other for serum extraction. Samples for serum extraction were left to clot for 30 min and then centrifuged at 3000 rpm for 10 min.

Participants were also asked to provide spot urine in the morning to measure the panel of urinary protein parameters (UA1MG, UACR, UIGG, and UTRF). All items were measured at Department of Clinical Laboratory of Beijing Tongren Hospital, Capital Medical University, in Beijing.

Serum hsCRP concentration and other biochemical items were measured on an AU5800 Automatic Analyzer from Beckman Coulter (USA). Serum hsCRP concentration was determined by means of Particle Enhanced Immunotransmission Turbidimetry method. Other biochemical items included FPG (fasting plasma glucose), BUN (blood urea nitrogen), SCr (serum creatinine), UA (uric acid), TP (total protein), ALB (albumen), Lpa (Lipoprotein a), TG (triglycerides), total cholesterol (TC), LDL-C (low density lipoprotein-cholesterol), and HDL-C (high density lipoprotein-cholesterol). Insulin (fasting) and C-peptide (fasting) were measured by means of chemiluminescent microparticle immunoassay method on ARCHITECT i2000SR (USA). The whole blood in K_2_ EDTA for HbA1c determination was measured by means of High Performance Liquid Chromatography with Ion Exchange method on HPL-723 G8 Automated Glycohemoglobin Analyzer (Japan).

All urine samples were spun at 2,000g for 5 minutes in a refrigerated centrifuge and were immediately transferred to new sample tubes and measured. The urinary creatine concentration was measured by means of sarcosine oxidase method on a DXC800 Automatic Analyzer from Beckman Coulter (USA), The concentrations of urinary A1MG, mAlb, IGG, and TRF were measured by means of scatter turbidimetry method on the Immage 800 Immunochemistry System from Beckman Coulter (USA). To account for variations in urine concentrations among individuals, all concentrations were expressed in units per gram urinary creatinine excretion. All the detection procedures strictly followed instructions.

### 2.4. Reference Interval for Serum hsCRP and Urinary Protein Parameters

In the present study, reference interval for hsCRP with the use of this laboratory detecting system is from 0 to 3 mg/l, that for UA1MG is from 0 to 10 mg/gCr, that for UACR is from 0 to 30 mg/gCr, that for UIGG is from 0 to 5 mg/gCr, and that for UTRF is from 0 to 1.5 mg/gCr. Furthermore, we defined hsCRP >3mg/L as high hsCRP, UA1MG>10 mg/gCr as high UA1MG, UACR>30mg/gCr as high UACR, UIGG>5mg/gCr as high UIGG, and UTRF >1.5mg/gCr as high UTRF.

### 2.5. Statistical Analysis

All statistical analysis was performed using SPSS 17.0 (SPSS Inc., Chicago, IL, USA). Two-tailed P value <0.05 was considered statistically significant. Categorical variables are expressed as frequencies. Continuous variables are described using mean ± standard deviation (SD) for normally distributed variables and medians with interquartile range (IQR) for nonnormally distributed data. According to hsCRP level, the participants were stratified into two groups with cut-off value of 3 mg/L.

Statistical differences for continuous variables in demographic and clinical characteristics between groups by gender and hsCRP were evaluated by Student's t test for normally distributed variables or Mann-Whitney U test for nonnormally distributed variables. The correlation of serum hsCRP levels and each urinary protein parameter (UA1MG, UACR, UIGG, and UTRF) was analyzed using the Pearson test. Also, we compared the distribution of hsCRP and urinary protein parameters in different groups according to each urinary protein parameter.

To determine the association of serum hsCRP and each urinary protein parameter (UA1MG, UACR, UIGG, and UTRF) in patients with T2DM, three models of linear regression and logistic regression were conducted for each explanatory variable, respectively. Model 1 was univariate analysis, model 2 was adjusted for age and gender, and model 3 was adjusted for variables that were significantly associated (p<0.05) in univariate analyses. In model 3, gender, age, and Scr were excluded as variables, because they entered the equation for eGFR calculation. Multivariate logistic regression models used the backward stepwise method, including variables that were significantly associated (p<0.05) in univariate analyses, and results were reported as odds ratios (OR) with 95% confidence intervals (95% CI). Nonnormally distributed variables, hsCRP, and urinary protein parameters (UA1MG, UACR, UIGG, and UTRF) were transformed using the natural logarithm (ln) before regression analysis.

## 3. Results

### 3.1. Clinical Characteristics of the Participants

The 520 patients consisted of 317 men and 203 women. Their biological parameters, biochemical and metabolic parameters, and urinary protein parameters were presented in Table 1. The mean age of the total participants was 58.59 years, and the mean duration of diabetes was 12.69 years. The mean body mass index (BMI) was 25.32 kg/m^2^.

As Table 1 showed, the median (IQR) level of serum hsCRP was 1.31(0.51-3.27) mg/L and higher hsCRP were found in female than in male (P=0.026). Regarding the urinary protein parameters, higher UIGG (P < 0.001) and higher UTRF (P=0.001) were found in female than in male, but there were no significant differences in UA1MG and UACR between female and male. For the other biochemical and metabolic parameters, higher TC and HDL-C and lower SCr, UA, and eGFR were found in female compared to male.

### 3.2. Comparisons of Clinical Variables according to Serum hsCRP Levels

The clinical parameters according to serum hsCRP levels were shown in Table 2. The participants were divided into two groups according to serum hsCRP levels. Patients with serum hsCRP ≤3 mg/L were classified as Low hsCRP group, and patients with serum hsCRP > 3 mg/L were classified as High hsCRP group. The median hsCRP levels in Low hsCRP group and High hsCRP group were 0.80mg/L and 5.50mg/L, respectively. The BMI (P < 0.001), UA1MG (P < 0.001), and UIGG (P=0.001) significantly increased in High hsCRP group. Higher FBG (P=0.003), higher TG (P=0.017), and higher HbA1C (P=0.024) were found in High hsCRP group compared to Low hsCRP group. The values of metabolic variables such as BUN, SCr, TC, and LDL-C did not differ significantly between the two groups. There were no significant differences in the UACR and UTRF between Low hsCRP group and High hsCRP group.

### 3.3. The Distribution of Serum hsCRP and the Panel of Urinary Protein Parameters in Patients with T2DM

We compared the distribution and difference of hsCRP and urinary protein parameters in different groups according to each urinary protein parameter. As Table 3 showed, 266 cases were classified as High UA1MG group (UA1MG >10mg/gCr) and 254 cases were classified as Low UA1MG group (UA1MG≤10mg/gCr). Higher UACR (P<0.001), higher UIGG (P<0.001), and higher UTRF (P<0.001) were found in High UA1MG group. It was worth noting that higher hsCRP (P=0.001) was only found in High UA1MG group. For hsCRP, there were no significant differences when cases were grouped according to UACR, UIGG, or UTRF, respectively. The UA1MG was significantly higher in High UACR group (UACR>30mg/gCr) in comparison with Low UACR group (UACR≤30mg/gCr) (P<0.001).

### 3.4. Association of Serum hsCRP and the Panel of Urinary Protein Parameters in Patients with T2DM by Regression Analysis

Multivariable linear and logistic regression analysis was carried out to assess whether hsCRP was independently associated with these urinary protein parameters (UA1MG, UACR, UIGG, and UTRF) by adjusting for likely confounders. As Table 4 showed, in multivariable linear regression analyses, lnhsCRP was positively associated with the lnUA1MG in all three models (adjusted *β* in model 3= 0.122, SE = 0.027, P<0.001). LnhsCRP was positively associated with the lnUIGG in model 1 (adjusted *β*=0.148, SE=0.038, P<0.001) and in model 2 (adjusted *β*=0.135, SE= 0.038, P<0.001) after adjustment for age and sex, but the association turned statistically insignificant after adjustment for confounders in model 3 (adjusted *β*= 0.039, SE=0.033, P=0.234). Similar significant findings were also found in lnUTRF. Although lnUTRF showed significance for association with lnhsCRP in model 1 (adjusted *β*=0.094, SE=0.039, P=0.016) and model 2 (adjusted *β*=0.081, SE=0.038, P=0.034), these were not statistically significant for association with lnhsCRP in model 3 (adjusted *β*=0.014, SE=0.035, P=0.694). For lnUACR, these were not statistically significant for association with lnhsCRP in all three models.

As Table 4 showed, when hsCRP≤3mg/L group was used as the reference, hsCRP>3mg/L group was independently associated with lnUA1MG in model 1 (adjusted *β*=0.452, SE=0.089, P<0.001), in model 2 (adjusted *β*=0.419, SE=0.087, P<0.001), and in model 3 (adjusted *β*=0.317, SE=0.078, P<0.001). For lnUIGG, hsCRP>3mg/L group was positively associated with the lnUIGG in model 1 (adjusted *β*=0.428, SE=0.112, P<0.001) and in model 2 (adjusted *β*=0.389, SE=0.110, P<0.001) after adjustment for age and sex, but the association turned statistically insignificant after adjustment for all confounders in model 3 (adjusted *β* = 0.162, SE= 0.094, P=0.085). It was worth noting that no positive associations were observed between hsCRP>3mg/L group and lnUACR in all models when hsCRP≤3mg/L group was used as the reference.

Furthermore, the analyses of multivariable logistic regression are presented in Table 5. Results showed that the High hsCRP group (hsCRP>3mg/L) was associated with increasing risk of high UA1MG (adjusted OR in model 3= 1.610, 95% CI 1.037–2.499, P=0.034). Similar significant findings were also found when we analyzed the relation between lnhsCRP and high UA1MG (adjusted OR in model 3= 1.366, 95% CI 1.147–1.626, P < 0.001). However, there were no significant associations between high hsCRP and high UACR, high UIGG, or high UTRF in model 2 and model 3 by analysis of multivariable logistic regression.

## 4. Discussion

In the present study, higher serum hsCRP levels were associated with a higher prevalence and severity of UA1MG in patients with T2DM. On the other hand, there were no associations of serum hsCRP levels with the presence of other urinary protein parameters (UACR, UIGG, and UTRF) by multivariable linear and logistic regression analysis. To the best of our knowledge, this is the first report on the relationship of serum hsCRP level and UA1MG in patients with T2DM.

Recent studies suggest that activation of innate immune system has an important role in the development of T2DM. It is known that hyperglycemia stimulates release of inflammatory cytokines from different types of cells and leads to induction and secretion of acute phase reactants such as hsCRP. Role of hsCRP, extremely sensitive but not specific marker of inflammation, in the pathogenesis of T2DM is a subject of extensive investigations [5]. HsCRP measurements have been used to be one of inflammation markers. The serum hsCRP levels in DM patients are known to be higher than that in healthy populations [11]. Tan et al. have shown that men with hsCRP concentration higher than 3 mg/L have 2.7 larger risk for development of T2DM compared to men whose CRP value was below 1 mg/L[26].

HsCRP is an acute-phase index of microinflammator response that can activate the complement system in the body and enhance the leukocyte phagocytosis by binding to the chromatin and can play a regulatory role by stimulating cell activation [2, 27]. Chronic endothelial inflammation is a major risk factor in the occurring of diabetic complications and has a pathogenic role in the progression of DN. The possible mechanisms may be that hsCRP may associate with DN through involving in the renal inflammation. Proinflammatory cytokines have been demonstrated as important factors in the development of microvascular diabetic complications, such as nephropathy [11, 28]. Previous findings have shown that hsCRP is an independent risk factor of obesity and T2DM and hs-CRP is closely related to DN [2, 29]. As we know, nuclear transcription factor-kappa B (NF-*κ*B) is active in inflammation and immune responses in human cells. NF-*κ*B signaling hs-CRP pathway is reported to be activated in DN and hsCRP inducing a series of proinflammatory cytokines through the NF-*κ*B-dependent mechanism [11, 30].

Results of this study showed that serum hsCRP was positively correlated with UA1MG by multivariate regression analysis. Higher hsCRP levels were found in higher UA1MG group, and higher UA1MG levels were found in higher hsCRP group. Urinary A1MG was recognized as a marker of proximal tubular dysfunction over two decades ago, but its use in clinical research studies was sparse until recent years. Urinary A1MG was also related to the duration, severity, and control of diabetes, indicating that it is a good marker of the severity of renal impairment in T2DM subjects [9, 31, 32]. Urinary A1MG levels were markedly elevated in diabetic patients when compared with control subjects and correlated directly with urinary albumin excretion and UACR and negatively with eGFR [33, 34], indicating the possible clinical application of urinary A1MG as a complementary marker for early detection of DN. In addition, the urinary excretion of A1MG was significantly higher in microalbuminuric in comparison with normoalbuminuric patients and controls, indicating tubular damage at an early stage of DN [33–35]. In another study, urinary A1MG was increased in 27.9% normoalbuminuric type 2 diabetic patients, indicating that urinary A1MG precedes the onset of albuminuria and may serve as a marker in early DN [36].

Clinically, urinary excess excretion of albuminuria, IGG, and TRF are usually markers of glomerular injury, while urinary A1MG is a sensitive marker for proximal tubular damage [9, 37–40]. The pathophysiology of albuminuria and tubulointerstitial damage are considered to be intertwined, where on one hand the reabsorption of increased amount of protein from the tubular lumen induces the proinflammatory and the profibrotic responses in tubular cells while, on the other hand, the damage of the proximal renal tubules alone can lead to albumin leak and consequently albuminuria [39, 41]. The detrimental effects of proteinuria appear to be mediated both at the glomerulus and in the proximal tubule, where the protein overload is toxic. In vitro, proximal tubular cells stimulated with serum proteins (albumin, IgG, and transferrin) produce a number of profibrotic and proinflammatory markers at the basolateral membrane including endothelin and IL-8, signaling for the recruitment of local macrophages. High molecular weight proteinuria has also been associated with proximal tubular cell apoptosis [42, 43]. Currently, there is a debate as to whether early DN in T2DM may be attributed to the glomerulus or to the proximal tubule (PT). It is assumed that albuminuria is caused primarily by impaired tubular uptake of intact albumin rather than by an increased leakiness of the glomerular filtration barrier. In previous works performed by us in normoalbuminuric patients with T2DM, we demonstrated that PT dysfunction precedes the occurrence of albuminuria and is dissociated from endothelial dysfunction [1, 34, 36].

In this study, it is interesting that UA1MG excretions had a significant relationship with serum hsCRP in patients with T2DM, but UACR, UIGG, and UTRF levels did not show significant correlation with hsCRP. Although it is not clear why serum hsCRP levels were positively correlated with the severity of UA1MG in T2DM, some possible explanations can be suggested. In response to autonomic dysfunction promoted by endothelial dysfunction, inflammation, and oxidative stress, the serum hsCRP level may be increased compensatorily. Physiologically, A1MG is involved in the defense against oxidative tissue damage [44]. Oxidative stress and increased inflammation play a key role in DN development. Chronic hyperglycemia enhances reactive oxygen species (ROS) production which causes the damage of the glomerular filtration barrier integrity, leading to albumin leakage, which can with ROS in the tubular ultrafiltrate further activate a variety of aberrant signaling pathways to cause overall renal function deterioration [9, 45].

Clinically, “microalbuminuria” is still accepted as an early biomarker of glomerular damage; some studies have also explored the relationship between hsCRP and UACR, but the conclusions are controversial [17]. In 2002, Stehouwer et al. [46] reported for the first time that CRP levels were associated with a subsequent increment in urinary microalbumin levels in patients with diabetes. Navarro et al. [47, 48] studied patients with type 2 diabetes and revealed that CRP levels were high in patients with microalbuminuria compared with those with normoalbuminuria. However, Schalkwijk et al. [49] reported they did not observe a significant difference in CRP levels between those with microalbuminuria and macroalbuminuria.

In our study, we found a significant relation of serum hsCRP with UA1MG, which suggested the proximal tubule is an important link in the development of DN. This association raises the possibility that renal tubular function defects precede the onset of microalbuminuria. Similar data have been provided by several studies performed in normoalbuminuric patients with type 2 DM, with increased levels of urinary alpha1-microglobulin [32, 36, 50], and showed that tubular functional defects precede the onset of albuminuria. Results of some studies showed that diabetic tubulopathy is an emerging entity that explains the occurrence of albuminuria in the early stages of diabetic nephropathy as a result of the impaired tubular reabsorption of albumin, rather than of its increased glomerular filtration [51–53]. Fu et al. [54] suggested that there is a link between the glomerular functional changes and the tubular damage: the glomerular hyperfiltration, which characterizes the early stages of diabetic nephropathy, could be a trigger for the proximal tubule dysfunction.

The strengths of the present study are that it is the first report on the association of serum hsCRP and UA1MG in patients with T2DM. Nevertheless, this study has several limitations. First, the sample size was small and from a single center. The cross-sectional nature of the study prevented the analysis of the associations between biomarkers and response to therapy. In addition, each patient's data came from the retrospective analysis of the patient's test at the time of admission, although the quality control of each index of the whole test system is within the controllable range, which may introduce some bias. Second, only a limited panel of biomarkers was assessed, and additional markers could be explored, such as tumor necrosis factor a (TNF-a), neutrophil gelatinase-associated lipocalin (NGAL), and interleukin (IL)-6[1, 17, 55]. Third, we did not have access to serum concentrations of A1MG, and, therefore, we cannot exclude the possibility that higher serum levels in susceptible individuals contributed to our observations. Finally, although we adjusted for multiple potential confounders, the possibility of residual confounding exists for our associations of urine UA1MG with hsCRP.

## 5. Conclusions

In conclusion, this present study suggests that serum hsCRP levels independently associate with UA1MG in patients with T2DM. The study highlighted the importance of hsCRP and UA1MG and is beneficial to the early diagnosis of renal injury. Future prospective studies with a larger sample size are required to explore the function of hsCRP and to establish a direct relationship between serum hsCRP levels and the UA1MG in development and treatment of T2DM.

## Figures and Tables

**Table 1:** Clinical and biological characteristics of the patients studied by gender.

Characteristics	Total	Male	Female	P value
*Biological parameters*				
Case number	520	317	203	
Age (year-old)	58.59±12.54	57.05±12.86	60.98±11.65	0.001
Duration of T2DM	12.69±5.50	12.69±5.45	12.68±5.60	0.852
BMI (kg/m^2^)	25.32±3.57	25.33±3.57	25.30±3.57	0.816
Systolic BP (mmHg)	131.03±16.66	130.22±15.71	132.30±18.01	0.657
Diastolic BP (mmHg)	77.39±10.92	77.73±10.93	76.87±10.89	0.517
*Biochemical and metabolic parameters*				
hsCRP (mg/L)	1.31(0.51-3.27)	1.08(0.50-3.03)	1.90 (0.58-3.87)	0.026
FPG (mmol/L)	7.56(5.91-9.59)	7.39 (5.98-9.10)	7.78 (5.81-10.17)	0.292
BUN (mmol/L)	5.60(4.60-6.80)	5.60(4.55-6.80)	5.50 (4.60-6.90)	0.425
SCr(*μ*mol/L)	65.60(53.56-77.50)	67.00(56.60-81.55)	59.20 (49.80-73.80)	<0.001
UA(*μ*mol/L)	339.80(286.73-404.03)	345.90(302.30-414.85)	316.30(277.80-381.14)	<0.001
TP(g/L)	64.90±5.29	64.58±5.16	65.39±5.47	0.168
ALB(g/L)	39.70(37.50-42.10)	39.70(37.40-42.05)	39.70 (37.60-42.10)	0.412
LPa (mg/dL)	11.92(5.10-26.75)	10.90 (4.30-25.30)	13.20 (6.10-27.50)	0.106
TG (mmol/L)	1.50(1.05-2.31)	1.47(1.03-2.34)	1.57 (1.11-2.27)	0.355
TC (mmol/L)	4.25(3.59-5.03)	4.22(3.52-4.87)	4.44 (3.72-5.46)	0.005
LDL-C (mmol/L)	2.42 (1.86-3.10)	2.39(1.84-2.99)	2.45 (1.92-3.23)	0.087
HDL-C (mmol/L)	1.03(0.86-1.25)	1.02(0.85-1.21)	1.07 (0.90-1.32)	0.009
Insulin (fasting)(uIU/mL)	7.40(4.50-12.50)	7.40(4.50-12.10)	7.40 (4.33-13.43)	0.807
C-peptide (fasting)(ng/mL)	1.17(1.10-2.60)	1.70(1.20-2.50)	1.70(1.10-2.68)	0.575
HOMA-IR	2.32(1.31-4.03)	2.23(1.31-3.98)	2.40 (1.30-4.14)	0.893
HbA1C(%)	8.84±1.91	8.91±1.94	8.71±1.88	0.240
eGFR (ml/min/1.73m^2^)	100.35(82.59-112.50)	105.70(92.71-117.63)	92.26 (74.79-101.70)	<0.001
*Urinary protein parameters*				
UA1MG (mg/gCr)	10.23 (5.85-19.94)	9.47(5.55-19.09)	11.44 (6.78-20.677)	0.134
UACR (mg/ gCr)	15.21(7.53-58.83)	14.42(7.10-54.84)	17.66 (8.35-61.88)	0.307
UIGG (mg/gCr)	6.59(3.90-13.50)	5.87(3.52-11.27)	8.08 (4.76-14.62)	<0.001
UTRF (mg/gCr)	3.20(2.00-6.00)	2.90(1.80-5.35)	3.90 (2.40-6.50)	0.001

Data are shown as mean±SD or median (IQR).

T2DM:type 2 diabetes mellitus; hsCRP: hypersensitive C-reactive protein; BMI:Body mass index; IQR: interquartile range; FPG: fasting plasma glucose; BUN: blood urea nitrogen; SCr: serum creatinine; UA: serum uric acid; TP: total protein; ALB: albumen Lpa: Lipoprotein a; TG: Triglycerides; TC:Total cholesterol; LDL-C: low density lipoprotein-cholesterol; HDL-C: high density lipoprotein-cholesterol; HOMA-IR:homeostasis model assessment-insulin resistance; HbA1C: glycosylated hemoglobin; eGFR: estimated glomerular filtration rate; UA1MG: Urinary alpha1-microglobulin to creatinine ratio; UACR: Urinary microalbumin to creatinine ratio; UIGG: Urinary immunoglobulin G to creatinine ratio; UTRF: Urinary transferrin to creatinine ratio; SD: standard deviation; IQR: interquartile range.

**Table 2:** Clinical and biological characteristics of the patients studied by serum hsCRP group.

characteristics	Low hsCRP group (hsCRP≤3mg/L)	High hsCRP group (hsCRP>3mg/L)	P value
*Biological parameters*			
Case number (Male/ Female)	379(238/141)	141(79/62)	
Age (year-old)	58.09±13.15	59.92±10.67	0.333
Duration of T2DM	13.04±5.34	11.75±5.90	0.090
BMI (kg/m^2^)	24.70±3.20	26.93±3.99	<0.001
Systolic BP (mmHg)	131.19±16.23	130.60±17.81	0.803
Diastolic BP (mmHg)	77.27±11.04	77.71±10.62	0.337
*Biochemical and metabolic parameters*			
hsCRP (mg/L)	0.80(0.40-1.49)	5.50 (4.12-7.96)	<0.001
FPG (mmol/L)	7.30(5.76-9.11)	8.13 (6.35-10.41)	0.003
BUN (mmol/L)	5.70(4.60-6.90)	5.40 (4.50-6.60)	0.131
SCr(*μ*mol/L)	66.30(54.80-77.90)	61.90(52.25-77.25)	0.314
UA(*μ*mol/L)	333.30(283.50-403.50)	353.50(301.15-408.70)	0.166
TP(g/L)	64.72±5.25	65.38±5.38	0.053
ALB(g/L)	40.10(37.90-42.70)	38.80(36.30-40.95)	<0.001
LPa (mg/dL)	11.70 (5.20-27.00)	12.60 (4.60-24.30)	0.716
TG (mmol/L)	1.47(1.00-2.21)	1.66 (1.18-2.48)	0.017
TC (mmol/L)	4.22(3.55-5.02)	4.39 (3.70-5.03)	0.234
LDL-C (mmol/L)	2.37(1.84-3.05)	2.57(1.96-3.19)	0.122
HDL-C (mmol/L)	1.06(0.88-1.29)	0.95(0.81-1.14)	0.001
Insulin (fasting)(uIU/mL)	6.75(4.10-11.88)	9.30(5.50-14.50)	0.002
C-peptide (fasting)(ng/mL)	1.60(1.10-2.40)	2.00(1.30-3.00)	0.001
HOMA-IR	2.07(1.19-3.76)	2.79(1.58-5.03)	0.004
HbA1C(%)	8.71±1.87	9.16±1.99	0.024
eGFR (ml/min/1.73m^2^)	100.19(82.60-113.17)	101.10(80.7-111.657)	0.533
*Urinary protein parameters*			
UA1MG (mg/gCr)	9.47(5.50-17.07)	14.22(7.26-30.99)	<0.001
UACR (mg/ gCr)	15.03(7.20-58.83)	19.47 (8.19-57.79)	0.371
UIGG (mg/gCr)	6.21(3.61-10.98)	7.81 (4.65-16.11)	0.001
UTRF (mg/gCr)	3.20(1.90-5.90)	3.50(2.20-8.25)	0.099

Data are shown as mean±SD or median (IQR).

T2DM:type 2 diabetes mellitus; hsCRP: hypersensitive C-reactive protein; BMI:Body mass index; IQR: interquartile range; FPG: fasting plasma glucose; BUN: blood urea nitrogen; SCr: serum creatinine; UA: serum uric acid; TP: total protein; ALB: albumen Lpa: Lipoprotein a; TG: Triglycerides; TC:Total cholesterol; LDL-C: low density lipoprotein-cholesterol; HDL-C: high density lipoprotein-cholesterol; HOMA-IR:homeostasis model assessment-insulin resistance; HbA1C: glycosylated hemoglobin; eGFR: estimated glomerular filtration rate; UA1MG: Urinary alpha1-microglobulin to creatinine ratio; UACR: Urinary microalbumin to creatinine ratio; UIGG: Urinary immunoglobulin G to creatinine ratio; UTRF: Urinary transferrin to creatinine ratio; SD: standard deviation; IQR: interquartile range.

**Table 3:** The comparison of hsCRP and each urinary protein parameter in different groups.

Group	Case number	hsCRP (mg/L)	UA1MG (mg/gCr)	UACR (mg/gCr)	UIGG (mg/gCr)	UTRF (mg/gCr)
*Group by UA1MG*						
UA1MG ≤10(mg/gCr)	254	1.00(0.49-2.80)	5.78(4.12-7.91)	9.04(4.98-19.87)	4.13(3.01-6.30)	2.30(1.50-3.33)
UA1MG >10(mg/gCr)	266	1.49(0.60-4.19)	19.25(14.09-32.85)	33.07(12.41-178.52)	11.26(7.08-26.45)	5.50(3.00-13.05)
P value		P=0.001	P<0.001	P<0.001	P<0.001	P<0.001
*Group by UACR*						
UACR≤30(mg/gCr)	334	1.29(0.50-3.19)	8.15(4.81-13.57)	9.07(5.61-14.56)	4.79(3.22-7.10)	2.40(1.60-3.70)
UACR>30(mg/gCr)	186	1.32(0.54-3.30)	18.54(10.35-35.86)	103.47(52.24-399.14)	16.68(9.17-45.42)	6.65(3.88-21.53)
P value		P=0.566	P<0.001	P<0.001	P<0.001	P<0.001
*Group by UIGG*						
UIGG ≤5(mg/gCr)	190	1.10(0.49-2.70)	5.26(3.92-8.37)	7.52(4.53-11.75)	3.40(2.66-4.13)	1.90(1.40-2.50)
UIGG >5(mg/gCr)	330	1.38(0.55-3.82)	15.22(9.13-28.10)	33.47(12.05-133.04)	10.09(6.95-20.53)	4.95(3.40-10.23)
P value		P=0.052	P<0.001	P<0.001	P<0.001	P<0.001
*Group by UTRF*						
UTRF ≤1.5(mg/gCr)	81	1.10(0.50-2.42)	4.30(2.89-8.22)	7.07(4.39-11.66)	2.66(2.09-3.67)	1.20(1.00-1.40)
UTRF >1.5(mg/gCr)	439	1.30(0.52-3.40)	12.41(7.10-22.94)	21.70(8.70-74.76)	7.68(4.77-15.37)	3.80(2.50-7.10)
P value		P=0.268	P<0.001	P<0.001	P<0.001	P<0.001

Data are shown as median (IQR)

hsCRP: hypersensitive C-reactive protein; UA1MG: Urinary alpha1-microglobulin to creatinine ratio; UACR: Urinary microalbumin to creatinine ratio; UIGG: Urinary immunoglobulin G to creatinine ratio; UTRF: Urinary transferrin to creatinine ratio.

**Table 4:** Multivariate linear regression for effects of serum hsCRP on each urinary protein parameter.

parameters	Model 1		Model 2		Model 3	
	*β* (SE)	P	*β*(SE)	P	*β* (SE)	P
*LnUA1MG *						
LnhsCRP	0.179 (0.030)	<0.001	0.169 (0.029)	<0.001	0.122(0.027)	<0.001
hsCRP≤3mg/L	Ref.		Ref.		Ref.	
hsCRP>3mg/L	0.452 (0.089)	<0.001	0.419 (0.087)	<0.001	0.317(0.078)	<0.001
*LnUACR *						
LnhsCRP	0.101 (0.057)	0.080	0.091 (0.057)	0.112	-0.012(0.052)	0.825
hsCRP≤3mg/L	Ref.		Ref.		Ref.	
hsCRP>3mg/L	0.207 (0.167)	0.216	0.175 (0.167)	0.294	-0.068(0.148)	0.649
*LnUIGG*						
LnhsCRP	0.148(0.038)	<0.001	0.135 (0.038)	<0.001	0.039(0.033)	0.234
hsCRP≤3mg/L	Ref.		Ref.		Ref.	
hsCRP>3mg/L	0.428 (0.112)	<0.001	0.389 (0.110)	<0.001	0.162(0.094)	0.085
*LnUTRF*						
LnhsCRP	0.094 (0.039)	0.016	0.081 (0.038)	0.034	0.014(0.035)	0.694
hsCRP≤3mg/L	Ref.		Ref.		Ref.	
hsCRP>3mg/L	0.251 (0.113)	0.027	0.212 (0.111)	0.057	0.036(0.099)	0.713

Model 1 univariate analysis

Model 2 adjusted for age and gender

Model 3 adjusted for duration of T2DM, systolic BP, FPG, BUN, TP, ALB, LDL-C, eGFR

hsCRP: hypersensitive C-reactive protein; UA1MG: Urinary alpha1-microglobulin to creatinine ratio; UACR: Urinary microalbumin to creatinine ratio; UIGG: Urinary immunoglobulin G to creatinine ratio; UTRF: Urinary transferrin to creatinine ratio.

**Table 5:** Multivariate logistic regression for effects of serum hsCRP on each urinary protein parameter.

parameters	Model 1		Model 2		Model 3	
	OR (95%CI)	P	OR (95%CI)	P	OR (95%CI)	P
*UA1MG >10(mg/gCr)*						
LnhsCRP	1.297 (1.128-1.491)	<0.001	1.282 (1.112-1.478)	0.001	1.366(1.147-1.626)	<0.001
hsCRP≤3mg/L	Ref.		Ref.		Ref.	
hsCRP>3mg/L	1.576 (1.064- 2.334)	0.023	1.498 (1.005- 2.234)	0.047	1.610(1.037-2.499)	0.034
*UACR>30(mg/gCr)*						
LnhsCRP	1.045 (0.904- 1.207)	0.552	1.032(0.892-1.194)	0.669	1.022(0.876-1.204)	0.797
hsCRP≤3mg/L	Ref.		Ref.		Ref.	
hsCRP>3mg/L	0.911 (0.594-1.399)	0.670	0.880 (0.571-1.354)	0.561	0.933(0.579-1.505)	0.777
*UIGG >5(mg/gCr)*						
LnhsCRP	1.165 (1.012- 1.342)	0.034	1.138(0.985-1.314)	0.078	1.140(0.977-1.331)	0.096
hsCRP≤3mg/L	Ref.		Ref.		Ref.	
hsCRP>3mg/L	1.510 (0.996-2.289)	0.052	1.407(0.922-2.147)	0.114	1.478(0.939-2.326)	0.091
*UTRF >1.5(mg/gCr)*						
LnhsCRP	1.117 (0.926-1.348)	0.247	1.072(0.883-1.301)	0.485	1.101(0.899-1.348)	0.352
hsCRP≤3mg/L	Ref.		Ref.		Ref.	
hsCRP>3mg/L	1.617 (0.901-2.903)	0.107	1.404(0.770-2.558)	0.268	1.547(0.831-2.881)	0.169

Model 1 univariate analysis

Model 2 adjusted for age and gender

Model 3 adjusted for duration of T2DM, systolic BP, FPG, BUN, TP, ALB, LDL-C, eGFR

hsCRP: hypersensitive C-reactive protein; UA1MG: Urinary alpha1-microglobulin to creatinine ratio; UACR: Urinary microalbumin to creatinine ratio; UIGG: Urinary immunoglobulin G to creatinine ratio; UTRF: Urinary transferrin to creatinine ratio.

## Data Availability

The data used in this article are displayed in tables. The raw data collected during an investigation are restricted in order to protect patient privacy. Requests for partial data that do not involve patient privacy, 12 months after publication of this article, will be considered for researchers who meet the criteria for access to confidential data by the corresponding author.
